# Detailed Clinical Characteristics, Interventions, and Long-Term Outcomes of Patients With Gastric Cancer Who Received the Best Supportive Care Without Any Anticancer Treatment

**DOI:** 10.1089/pmr.2023.0066

**Published:** 2023-12-07

**Authors:** Yohei Arihara, Ryo Shibuya, Michihiro Ono, Norito Suzuki, Ginji Omori, Yuki Ikeda, Hajime Nakamura, Michiko Yamada, Tomoyuki Abe, Kohichi Takada, Masahiro Maeda

**Affiliations:** ^1^ Department of Gastroenterology, Steel Memorial Muroran Hospital, Hokkaido, Japan.; ^2^ Department of Medical Oncology, Sapporo Medical University School of Medicine, Hokkaido, Japan.; ^3^ Department of Pancreatobiliary Medicine, Steel Memorial Muroran Hospital, Hokkaido, Japan.

**Keywords:** aged, gastric cancer, health services for the aged, palliative care, supportive care

## Abstract

**Background:**

Due to the lack of studies, the long-term prognoses of unfit patients with gastric cancer (GC) who did not receive any aggressive cancer treatment (best supportive care [BSC] cases) remain unclear, especially for those with potentially curable GC. We conducted this observational study to capture the real-world data of characteristics and outcomes for BSC cases.

**Method:**

Consecutive clinical records of patients with GC diagnosed at Steel Memorial Muroran Hospital from January 2017 to December 2021 were analyzed.

**Result:**

Of 481 patients diagnosed with GC, 91 (18.9%) were BSC cases. The median overall survival (OS) was 12.4, 8.3, and 2.5 months for clinical stage (cStage) I, II–III, and IV, respectively. Patients with potentially curable GC (cStage I–III) had significantly longer OS than those with incurable disease (cStage IV), with a hazard ratio for death of 0.29 (95% confidence interval: 0.18–0.47).

**Conclusion:**

Our report provides useful information for decision-making for unfit patients with GC in daily clinical practice.

## Background

Gastric cancer (GC) is one of the leading causes of cancer-related death worldwide. With multimodality treatment strategies, including curative surgery, chemotherapy, and immune checkpoint inhibitors (ICIs), the prognosis of patients with GC has been improving in the last few decades.^1–3^ In addition, due to the decreasing invasiveness of treatment and appropriate medical management, evidence supporting the benefits of anticancer treatment for elderly GC patients has accumulated. For example, Endo et al. reported a favorable prognosis of patients with GC aged 85 years or older, who underwent surgery, compared to those who received only the best supportive care (BSC).^4^ Hayashi et al. reported the benefit of chemotherapy for elderly Stage IV GC patients.^5^ Further, Paderi et al. reported noninferior tolerability and effectiveness of ICIs for elderly compared to younger patients.^6^ Based on these data, medical practitioners in developed countries tend to recommend aggressive anticancer treatment for elderly patients with GC. These treatment strategies are generally acceptable and need not be avoided just because of the patient's advanced age. However, we sometimes encounter patients with GC who are unsuitable for any anticancer treatment (even with reduced intensity) because of their frailty, dementia, or extremely advanced age. In addition, due to the lack of studies that focused on these populations, the long-term prognoses of unfit patients with GC who did not receive any aggressive cancer treatment remain unclear, especially for those with potentially curable disease (Stage I–III). Therefore, clinicians sometimes have trouble answering unfit patients or their caregivers when asked questions such as “How long will I live without any anticancer treatment?”

Hence, we conducted this observational study to capture the real-world data of baseline characteristics, supportive care, and long-term outcomes for unfit patients with GC who had not received any aggressive cancer treatment.

## Methods

### Study design

In this retrospective single-center observational study, we collected consecutive clinical records of patients diagnosed with GC at Steel Memorial Muroran Hospital (SMMH, an Acute Care Center Hospital in Hokkaido, Japan) from January 2017 to December 2021. Of those, we analyzed all patients who did not receive any aggressive anticancer treatment (BSC cases). Their baseline characteristics, interventions performed, and survival rates were evaluated.

### Outcomes, clinical evaluation, and statistical analysis

The primary outcome of this study is overall survival (OS). OS was calculated from the date of diagnosis of GC until death, analyzed using the Kaplan–Meier method, and compared with the log-rank test. The date and cause of death were identified from the medical records. Patients were followed until death or the data cutoff date of December 31, 2021.

The indication of anticancer therapy for elderly patients should be determined after conducting a geriatric assessment.^7^ Therefore, we assessed the patient's Age-Adjusted Charlson Comorbidity Index (ACCI, to assess the severity of comorbidities)^8^ and the G-8 score (screening tool for geriatric assessment),^9^ as a part of daily practice. Physical and laboratory findings were also collected at the time of diagnosis with GC. At the time of data collection, we retrospectively evaluated the clinical stage (cStage) of GC, based on the Union for International Cancer Control (UICC)-TNM 8th edition. Chi-squared test and two-sided Student's *t*-test were used to compare patient characteristics and laboratory findings between groups. *p*-Values <0.05 were considered statistically significant. Statistical analyses and graph visualization were performed using GraphPad Prism (v.9.5.1; GraphPad Software, SanDiego, CA, USA).

## Result

### Clinical characteristics

During the above period, 481 patients were diagnosed with GC in SMMH. Of those, 91 (18.9%) were BSC cases with a median age of 85 (range: 46–96) years; 75.8% were male. On the one hand, 89.1% of the participants were histologically diagnosed as GC; on the other hand, 10.9% were diagnosed only with radiological findings and tumor markers. The patients were classified as cStage I (17.6%), II–III (25.3%), and IV (57.1%). One-third (34.1%) of all participants had cognitive dysfunction, and 65.9% revealed poor performance status (Eastern Cooperative Oncology Group performance status; PS ≥2). The median body mass index (BMI) of the patients was 20.2 kg/m^2^ (range: 14.0–39.9). The median hemoglobin (Hb), serum albumin (Alb), and C-reactive protein (CRP) levels of the entire cohort were 10.3 g/dL, 3.0 mg/dL, and 1.3 mg/dL, respectively. BSC cases appeared to be at a very high risk of mortality/adverse event by aggressive cancer treatment with a median ACCI score of 7 and a G-8 score of 7. The background comparison of each cStage showed no significant differences in baseline BMI, Hb, Alb, CRP, and G-8 scores. However, compared to cStage IV group, cStage I–III had more elderly patients, patients with higher ACCI, and those taking more medications.

In general, concomitant disease or not adequate organ function could be the key reason for BSC. However, not only for these reasons but each patient also had specific and entangled background involved in decision-making. The most critical reasons for aggressive cancer treatment not being indicated were as follows: extremely old age (29.6%), dementia (10.9%), poor PS or organ dysfunction (16.4%), and the patient's own wishes (34.1%). These results may reflect patients' values and preferences and not directly reflect baseline characteristics. Detailed patient characteristics are shown in Table 1.

**Table 1.** Patient Characteristics

	All patients (***N*** = 91)	cStage I–III (***n*** = 39)	cStage IV (***n*** = 52)	Comparison cStage I–III versus IV
Age median (range), years	85 (46–96)	88 (68–95)	83.5 (46–96)	*p* = 0.009
Male sex, *n* (%)	69 (75.8)	29 (74.4)	40 (76.9)	*p* = 0.777
ECOG PS, *n* (%)				
0	7 (7.7)	6 (15.4)	1 (1.9)	*p* = 0.750 (% of PS ≥2)
1	24 (26.4)	8 (20.5)	16 (30.8)	
2	23 (25.3)	12 (30.8)	11 (21.2)	
3	22 (24.2)	5 (12.8)	17 (32.7)	
4	15 (16.5)	8 (20.5)	7 (13.5)	
BMI, median (range), kg/m^2^	20.2 (14.0–39.9)	20.6 (14.2–26.9)	20.0 (14.0–39.9)	*p* = 0.970
ACCI, median (range)	7 (4–17)	9 (5–17)	7 (4–12)	*p* = 0.003
G-8, median (range)	7 (1–14.5)	7.5 (1.5–14.5)	7 (1–14)	*p* = 0.311
Cognitive dysfunction, *n* (%)	31 (34.1)	16 (41.0)	15 (28.8)	*p* = 0.225
Comorbidities, *n* (%)				
Chronic heart failure	11 (12.1)	7 (17.9)	4 (7.7)	*p* = 0.195
Cerebrovascular disease	11 (12.1)	6 (15.4)	5 (9.6)	*p* = 0.519
Chronic pulmonary disease	8 (8.8)	4 (10.3)	4 (7.7)	*p* = 0.721
Diabetes	16 (17.6)	8 (20.5)	8 (15.4)	*p* = 0.585
Hypertension	37 (40.7)	19 (48.7)	18 (34.6)	*p* = 0.201
Use of anticoagulants, *n* (%)	13 (14.3)	8 (20.5)	5 (9.6)	*p* = 0.142
Use of antiplatelets, *n* (%)	17 (18.7)	6 (15.4)	11 (21.2)	*p* = 0.485
Site of primary tumor, *n* (%)				
Upper	15 (16.5)	5 (12.8)	10 (19.2)	*p* = 0.686
Middle	47 (51.6)	19 (48.7)	28 (53.8)	
Lower	21 (23.1)	10 (25.6)	11 (21.2)	
N/A	8 (8.8)	5 (12.8)	3 (5.8)	
Pathological confirmation, *n* (%)	81 (89.0)	34 (87.2)	47 (90.4)	*p* = 0.629
Laboratory findings				
Hemoglobin, median (range), g/dL	10.3 (4.4–18.6)	9.9 (4.4–14.7)	10.4 (4.7–18.6)	*p* = 0.175
Alb, median (range), g/dL	3.0 (1.2–4.3)	3.1 (1.6–4.3)	3.0 (1.2–4.3)	*p* = 0.339
CRP, median (range), mg/dL	1.3 (0.0–20.3)	0.67 (0.0–16.8)	1.86 (0.1–20.3)	*p* = 0.307

ACCI, age-adjusted Charlson Comorbidity Index; Alb, albumin; BMI, body mass index; CRP, C-reactive protein; cStage, clinical stage; ECOG PS, Eastern Cooperative Oncology Group performance status; N/A, not available.

### Long-term outcomes

**FIG. 1.**
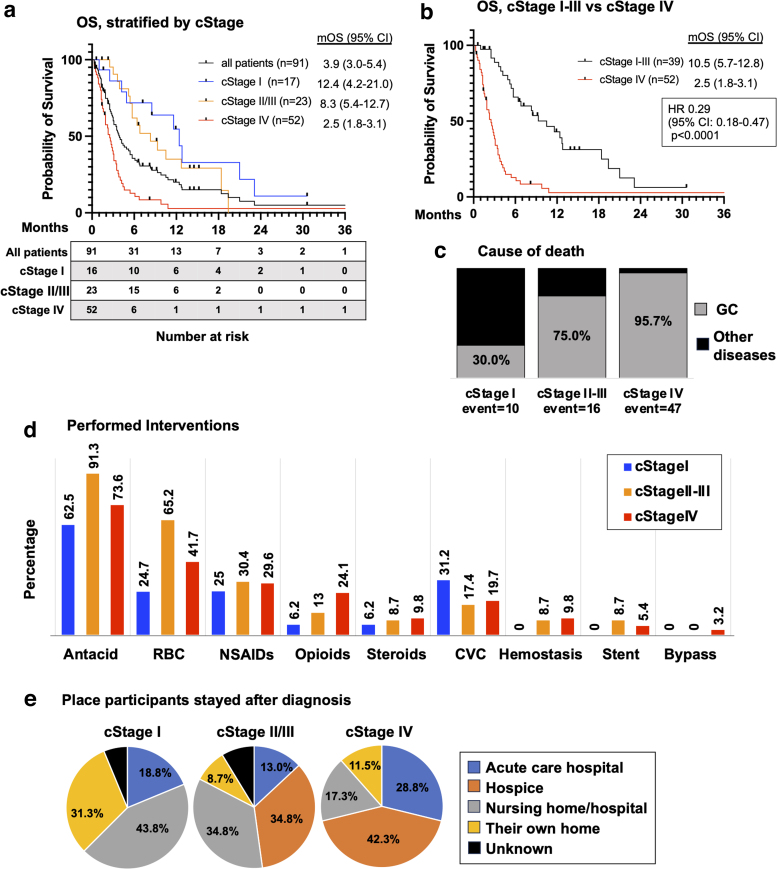
OS, cause of death, and performed interventions. **(a, b)** The Kaplan–Meier curves for OS of all patients, stratified by cStage. A comparison of each group was performed with the log-rank test. **(c)** The cause of death stratified by each cStage. **(d)** The intervention after diagnosis of GC, stratified by each cStage. **(e)** The place where the participants mostly stayed after diagnosis of GC. OS, overall survival; mOS, median overall survival; cStage, clinical stage; HR, hazard ratio; CI, confidence interval; GC, gastric cancer; RBC, red blood cell transfusion; NSAID, nonsteroidal anti-inflammatory drugs; Steroids, corticosteroids; CVC, central venous catheter insertion; Hemostasis, endoscopic hemostasis; Stent, endoscopic gastrointestinal stent placement; Bypass, gastro-jejunal bypass.

During the observational period, 73 patients (80.2%) died. The median OS for all patients was 3.9 months (95% confidence interval [CI]: 3.0–5.4), while the median OS was 12.4, 8.3, and 2.5 months for cStage I, II–III, and IV, respectively (Fig. 1a). Patients with potentially curable GC (cStage I–III) had significantly longer OS (median: 10.5 months) than those with incurable disease (cStage IV), with a hazard ratio for death of 0.29 (95% CI: 0.18–0.47, *p* < 0.0001) (Fig. 1b). Further, the long-term prognosis for BSC cases was shown to worsen as the stage of GC increased. Similarly, the percentage of death by GC in a total number of deaths also increased with increasing stage: 30%, 75%, and 95.7% for cStage I, II–III, and IV, respectively (Fig. 1c). Other than GC, pneumonia (33.3%) and heart failure (22.2%) were the most common cause of death.

### Therapeutic interventions

Therapeutic interventions performed for BSC cases included antacids (73.6%), red blood cell transfusions (41.8%), and central venous catheter insertion (19.8%) (Fig. 1d). After diagnosis, early-stage patients were more likely to prefer to stay where they had lived (nursing homes or their own house), while more advanced-stage patients chose hospice (Fig. 1e).

## Discussion

Here, we have reported the detailed clinical information of patients with GC who received BSC without any anticancer treatment. In brief, the median OS of BSC cases with GC was ∼1 year for cStage I, 8 months for cStage II–III, and <3 months for cStage IV. The mortality rate due to GC increased with increasing stage, suggesting that even in BSC cases, the more advanced the stage, the more GC adversely affects the life expectancy of the patients. Also, many of the patients with early-stage GC died from diseases other than GC, such as heart failure and pneumonia. This suggests that the prognosis of comorbidities must be carefully considered when making treatment decisions for elderly or frail patients with cancer. These results are very useful information when explaining the disease and its expected natural course to patients and their families in daily clinical practice.

Our report provoked some additional clinical questions: (1) Are there any baseline characteristics that can predict the prognosis of BSC cases? (2) Are there any clinical interventions that can improve the prognosis of these patients? To verify the results of this study and to answer the questions raised, we are now preparing a multicenter observational cohort study with a higher volume of subjects.

## Abbreviations Used

ACCI: adjusted Charlson Comorbidity Index
Alb: albumin
BSC: best supportive care
BMI: body mass index
CI: confidence interval
CRP: C-reactive protein
cStage: clinical stage
ECOG PS: Eastern Cooperative Oncology Group performance status
GC: gastric cancer
Hb: hemoglobin
ICI: immune checkpoint inhibitor
OS: overall survival
